# Motivations for Contributing to Health-Related Articles on Wikipedia: An Interview Study

**DOI:** 10.2196/jmir.3569

**Published:** 2014-12-03

**Authors:** Nuša Farič, Henry WW Potts

**Affiliations:** ^1^UCLCentre for Health Informatics & Multiprofessional Education (CHIME)University College LondonLondonUnited Kingdom

**Keywords:** motivation, writing, social media, wikis, Wikipedia, consumer health information

## Abstract

**Background:**

Wikipedia is one of the most accessed sources of health information online. The current English-language Wikipedia contains more than 28,000 articles pertaining to health.

**Objective:**

The aim was to characterize individuals’ motivations for contributing to health content on the English-language Wikipedia.

**Methods:**

A set of health-related articles were randomly selected and recent contributors invited to complete an online questionnaire and follow-up interview (by Skype, by email, or face-to-face). Interviews were transcribed and analyzed using thematic analysis and a realist grounded theory approach.

**Results:**

A total of 32 Wikipedians (31 men) completed the questionnaire and 17 were interviewed. Those completing the questionnaire had a mean age of 39 (range 12-59) years; 16 had a postgraduate qualification, 10 had or were currently studying for an undergraduate qualification, 3 had no more than secondary education, and 3 were still in secondary education. In all, 15 were currently working in a health-related field (primarily clinicians). The median period for which they have been an active editing Wikipedia was 3-5 years. Of this group, 12 were in the United States, 6 were in the United Kingdom, 4 were in Canada, and the remainder from another 8 countries. Two-thirds spoke more than 1 language and 90% (29/32) were also active contributors in domains other than health. Wikipedians in this study were identified as health professionals, professionals with specific health interests, students, and individuals with health problems. Based on the interviews, their motivations for editing health-related content were summarized in 5 strongly interrelated categories: education (learning about subjects by editing articles), help (wanting to improve and maintain Wikipedia), responsibility (responsibility, often a professional responsibility, to provide good quality health information to readers), fulfillment (editing Wikipedia as a fun, relaxing, engaging, and rewarding activity), and positive attitude to Wikipedia (belief in the value of Wikipedia). An additional factor, hostility (from other contributors), was identified that negatively affected Wikipedians’ motivations.

**Conclusions:**

Contributions to Wikipedia’s health-related content in this study were made by both health specialists and laypeople of varying editorial skills. Their motivations for contributing stem from an inherent drive based on values, standards, and beliefs. It became apparent that the community who most actively monitor and edit health-related articles is very small. Although some contributors correspond to a model of “knowledge philanthropists,” others were focused on maintaining articles (improving spelling and grammar, organization, and handling vandalism). There is a need for more people to be involved in Wikipedia’s health-related content.

## Introduction

Wikipedia is a free online encyclopedia created through the collaborative efforts of volunteers. As an open wiki, anyone can freely add to, modify, or delete its contents. It has become a major source of health-related information for health care professionals, students, patients, and the general public.

Wikipedia is the largest online encyclopedia with more than 26 million articles in 287 different languages and more than 4 million in the English-language Wikipedia [1]. It currently ranks as the sixth most-visited site on the Internet [2], attracting over 365 million unique visitors monthly or 29.5% of global Internet consumers [3,4]. Seeking health information online is now commonplace and widespread globally [5-11]. As of March 2013, the English-language Wikipedia contained more than 28,216 medical articles [12] and is a prominent repository of online health information. When health terms are searched in popular search engines such as Google and Yahoo, Wikipedia appears in the top 10 results 71%-85% of the time [13]. Wikipedia’s global popularity as an online health resource has also been observed among physicians, with 70% reporting using it in 1 study [14].

Wikipedia has attracted controversy around the reliability of its entries [15]. A comparison of science-related topics with Encyclopaedia Britannica indicated a comparable error rate [16]. Czarnecka-Kujawa and colleagues [17] found Wikipedia’s medical specialty entries were comprehensive (compared with ICD-9/10) and had moderate reliability. The risks associated with misinformation have raised the standards of control on Wikipedia where there are numerous policies, guidelines, and collaborative systems in place to ensure the quality of information [18]. The focus of these has been on biographical articles where there is a threat of libel litigation rather than on health-related articles, but there are many specific guidelines on medical topics. Although the reliability of Wikipedia is of great importance, it is not the primary field of enquiry for this study. We suggest that the debate around the reliability of information is too simple. Equally accurate articles can have different focuses, different styles of writing, and so on, all which may affect how useful articles are for different audiences and how different audiences use articles. The content of Wikipedia articles ultimately depends on those who contribute to Wikipedia (Wikipedians) and their reasons for doing so, and this is our focus.

There are 18 million registered accounts on Wikipedia. Several studies have examined the characteristics of Wikipedians: they are more familiar with the topics they edit than average Internet users [19] and more often male [20-22]. A 2011 Wikimedia survey of more than 5000 Wikipedians found an average age of 28 years, with 61% having a university degree, 18% a Master’s, and 8% a Doctoral degree. Previous studies examined culturally bound differences in Wikipedia contribution [23] and found Wikipedians score significantly lower on agreeableness, conscientiousness, and openness in the Big Five personality traits compared to non-Wikipedians [24]. However, characteristics of those specifically editing health-related pages have not been described.

A number of studies have examined knowledge-sharing intention and behavior [25-27]. Knowledge-sharing behavior has traditionally been studied in an organizational context; recently its principles have been applied to electronic networks [28]. This study assumed that contribution to Wikipedia can be classified as knowledge-sharing behavior because individuals largely engage by contributing what they know to specific pages.

Research on knowledge-sharing behavior has revealed 2 classes of motivations. These are intrinsic motivations, such as internal feeling of enjoyment and satisfaction [29], and extrinsic or goal-directed motivations, such as obtaining a reward and reputation [30]. The expectation of either obtaining the internal gratification or extrinsic returns may motivate individuals. However, Wikipedia poses a departure from conventional modes of knowledge-sharing behavior. Wikipedia is the prime example of a “commons-based peer production” model [31,32]. Wikipedians do not receive monetary or formal awards for their voluntary contributions and their unconventional modes of engagement have received scholarly attention [33,34]. Therefore, conventional motivational theory may be circumscribed in the context of Wikipedia and it is individual personality differences that affect how the information is produced and used. For example, Kuznetsov [35] found that Wikipedians are motivated by a process of interrelated value systems, such as altruism, reciprocity, community, autonomy, and reputation. It would be reasonable to surmise that in Wikipedia the exchange of knowledge is not based on interpersonal relationships (ie, intrinsic, extrinsic gains), but on the relative merit and importance one assigns to the context in which knowledge is shared [20,30,35].

Recognizing the limitations of conventional motivational theory, Leonard et al [36] proposed a model of self-concept motivations: the individuals are motivated to perform a behavior based on their inherent standards (internal self-concept) or standards that are in accordance with a reference group (external self-concept). The individual’s motivations stems either from meeting a set of perceptions of the self (ie, of their own values and competencies) or the perceptions of the ideal-self (ie, of values, competencies, and success of the reference group). Applying this to Wikipedia, Yang and Lai [20,30] observed that internal self-concept-based motivation is the chief motivation predictor in knowledge-sharing intention and behavior. Individuals were most likely to share knowledge in Wikipedia due to the confidence in their capabilities, affirmed by the concept of self-efficacy. Studies on organizational knowledge sharing have shown that self-efficacy is the crucial predictor of knowledge-sharing intention and behavior [25,37]. Yang and Lai [30] also revealed that the quality of the information and the quality of the information system yielded a positive attitude toward Wikipedia. Prior research of individual behavior has demonstrated that individual attitudes are good predictors of behavior and have been found to be crucial in knowledge-sharing intention and behavior [21,26,27,35,38].

These prior studies suggest the motivations behind Wikipedia contributions, highlighting that motivated behavior rarely pertains to a single motivation [39]. Considering the lack of data on motivations of Wikipedia contributors, a growing number of health-related queries online and Wikipedia’s status as a prominent resource for health information, it is important to answer the question of who contributes to the health-related Wikipedia pages and why.

## Methods

### Design

We employed a cross-sectional design. The study was conducted between May to September 2012. The recruited sample were Wikipedia users with an editing history in health-related entries in the English-language Wikipedia. The first part of the study used a questionnaire, whereas the second part used semi-structured interviews.

### Ethics

The study was approved by the University College London (UCL) Ethics Committee and the Wikimedia Foundation Research Committee.

### Article Sampling

To sample contributors of health-related articles, we first generated a sample of health-related articles on Wikipedia. It is difficult to sample from all health-related articles on Wikipedia. Wikipedia articles are sorted into categories, but this task is done in the same way as all Wikipedia editing and is incomplete. Articles vary in size, importance, and how often they are accessed. Defining what constitutes being health-related is also difficult, with many marginal cases.

An earlier study on Wikipedia extracted health-related keywords from 3 indexes of the online health service websites, namely MedlinePlus, National Health Service (NHS) Direct Online, and the National Organization of Rare Disorders (NORD), the last to oversample rarer conditions [12]. Their lists of keyword phrases consisted of 1726 items for MedlinePlus, 966 items for NHS Direct Online, and 1173 items for NORD. We randomly selected 11 keyword phrases from each of these lists. These were entered into the search box on Wikipedia and the best matching article chosen. In addition, the study used articles listed under the category of Selected Articles on the Wikipedia Portal Medicine [40], constituting a set of articles the Wikipedia community have chosen to highlight as being of high quality and interesting. The 11 most recently edited articles were chosen from the selected article list from between July 11-25, 2012. The final sampled articles list is shown in Table 1.

Having produced a sample of articles, a sample of contributors was produced by selecting the most recent 5 contributors for each article listed under the “history” tab.

**Table 1:** The sampled articles list for each of the medical databases and Wikipedia Portal Medicine. For the first 3 columns, the first term is the keyword phrase selection, whereas the bracketed term refers to the Wikipedia article name if it was not the same.

MedlinePlus	NHS Direct	NORD	Wikipedia Portal Medicine
Barrett’s esophagus	Contact dermatitis	Very long chain acyl CoA dehydrogenase deficiency (very long-chain acyl-coenzyme A dehydrogenase deficiency)	Asthma
Menopausal hormone therapy (hormone replacement therapy (menopause))	Oral thrush (oral candidiasis)	Fiber type disproportion (congenital fiber type disproportion)	Insulin
Living wills (advance health-care directive)	Epidermolysis bullosa	Congenital fibrodysplasia ossificans progressive (fibrodsysplasia ossificans progressiva)	Helicobacter pylori
AMD (muscular degeneration)	Indigestion	Pancreatic islet cell tumor (pancreatic cancer)	Forensic facial reconstruction
Methamphetamine	Artificial insemination	Dubin Johnson Syndrome	Metabolism
Osteonecrosis (avascular necrosis)	Rectal examination	L1 syndrome (MASA syndrome)	Influenza
ERT (hormone replacement therapy)	Rheumatic fever	Leukodystrophy, metachromatic (metachromatic leukodystrophy)	Sexually transmitted disease
Staphylococcal infections (*Staphylococcus*)	Vitiligo	Cerebral palsy	Female hysteria
Arthrography	Nasal polyps (nasal polyp)	Irritable bowel syndrome	Vacutainer
Arm injuries and disorders (median nerve palsy)	Bulimia	Mesothelioma	Nutrition
Implantable defibrillators (implantable cardioverter defibrillator)	Psychotherapy	Cataracts (cataract)	2007 Bernard Mathews H5N1 outbreak

**Figure 1:**
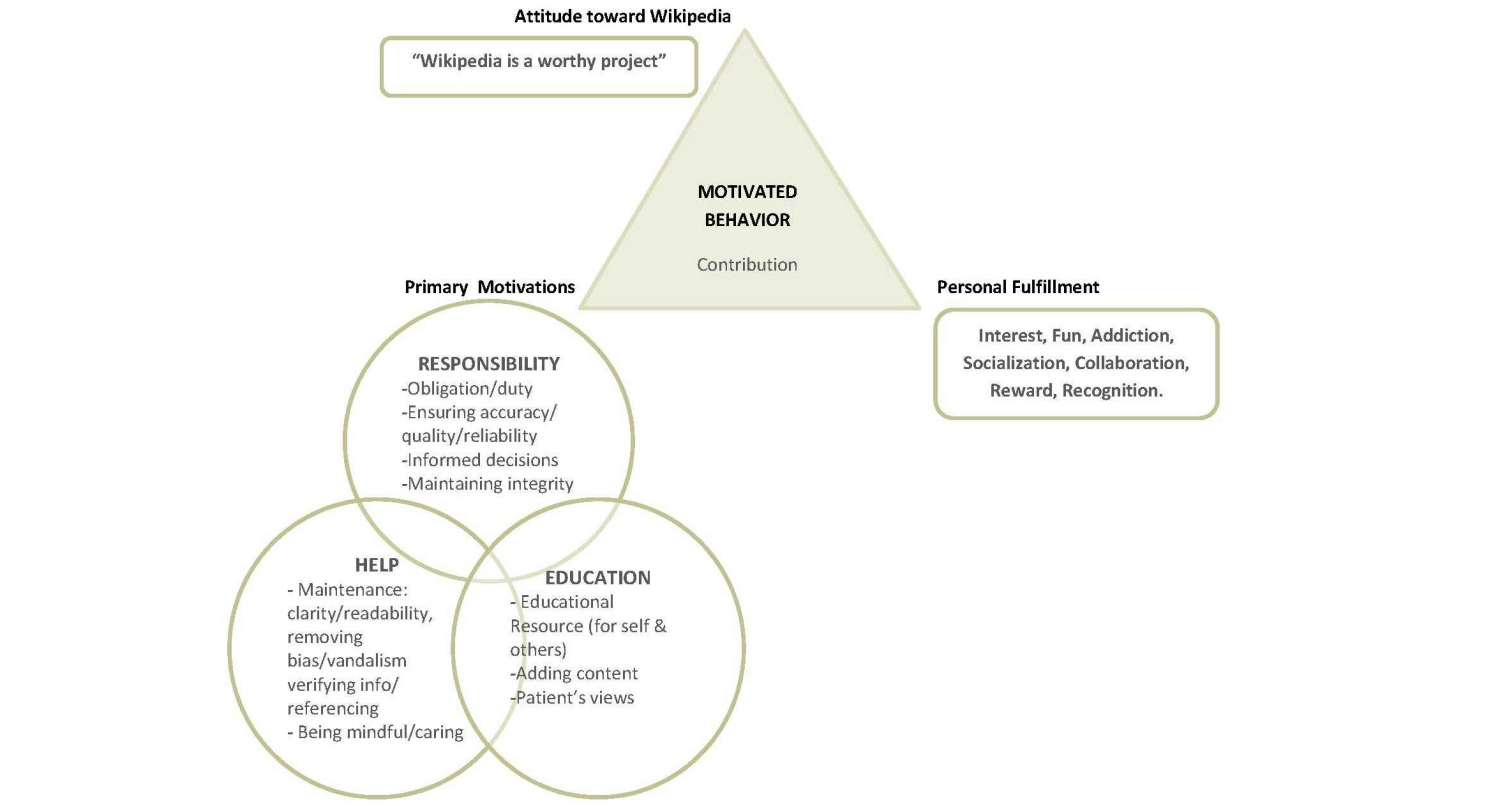
The motivational model.

Wikipedia users may register and create an account or they may edit articles without registering (or without logging into their registered account), in which case their edits are shown as coming from an Internet Protocol (IP) address. We included both registered (account) and nonregistered (IP address) contributors. Bots (automated or semiautomated software tools) were excluded. Every account and every editing IP address has or can have a Talk page, where a standardized invitation message was placed. NF set up a Wikipedia account for the purposes of recruitment [41].

A total of 44 articles were selected, which could have yielded up to 220 contributors to contact. In practice, many individuals came up more than once in the sampling frame, in which case, accounts not previously contacted were chosen until 220 different accounts were contacted.

The invitation message included brief information about the purpose of the study and the selected members were asked to follow a link that took them to NF’s Wikimedia Commons page [41] containing instructions for participation and information about the study. The instructions asked participants to complete a Web-based questionnaire by clicking on the provided hyperlink. This was run through UCL’s Opinio system [42].

### Questionnaire

A 16-item questionnaire included questions referring to participants’ characteristics, such as age, country of residence, employment, education, and Wikipedia editing history (including types of edits, editing in other languages, and number of health-related pages edited). The survey also included the question “What are your main motivations for editing health-related pages on Wikipedia?” with a free-text response. The penultimate item asked participants whether they would be willing to be interviewed and the final item asked for contact details (Wikipedia username or email address). A total of 32 complete survey responses were received (response rate 14.5%). Of these, 91% (29/32) agreed to be interviewed.

### Participants

All participants were registered Wikipedians who had previously edited at least 1 health-related page on the English-language Wikipedia. A total of 32 Wikipedians (31 male and 1 female) volunteered to participate and completed the questionnaire. Due to time constraints, only 17 participants were interviewed through Skype, via email, or in person at UCL (in London). The reason for a much smaller proportion of participants interviewed than indicated is that they did not respond when contacted to be interviewed. All interviewees returned a signed and dated copy of the informed consent form. The age of the questionnaire respondents ranged from 12 to 59 years with a mean of 37 (SD 13) years. The mean age of the interview sample was 40 years.

### Interviews

#### Overview

Two different semi-structured interview formats were used, but using the same interview schedule, developed around topics exploring participants’ personal characteristics and individual motivations around editing Wikipedia. The questions were open-ended and were presented in the same order in both interview settings. The interview guides were developed by eliciting information around the topic of interest (ie, experience and motivations for editing) [43]. The interviews followed a general-to-specific approach and interviews were piloted before full use and slight amendments made following a reflexivity exercise [44].

#### Interview Schedule

The first interview schedule consisted of 25 questions that participants were asked to answer in a written format and return the completed answers to the researcher via email. The second schedule allowed participants to respond to the questions directly either face-to-face or via Skype. Responses were recorded with a digital voice recorder. Both schedules incorporated questions (described subsequently). In most cases, the interview answers prompted issues requiring further exploration, which was done in an unstructured manner. If a participant responded by email, additional questions were sent via email. Face-to-face and Skype interviews lasted 30-120 minutes. Written responses contained from 969 to 3475 words. The list of questions used in the interview were:

1. What do you do and what are your specific interests?
2. What propelled you to start editing Wikipedia health-related pages?
3. Why did you edit the specific health-related page(s)?
4. Are your interests related to your Wikipedia edits?
5. Why do you edit with an account vs nonaccount?
6. What type of vandalism do you revert and why?
7. Do you have a particular group of readers in mind when you are editing health-related Wikipedia pages?
8. What are your main motivations for editing health-related content on Wikipedia?
9. Please talk about your reasons for editing health-related Wikipedia pages and comment on whether the reasons are entirely personal or driven by any external factors (such as a group of people, organization, or a particular individual)?
10. Are there any factors you can think of that would impact your motivations for editing health-related content on Wikipedia?
11. Some people say that one of their main motivations is to promote collaboration between patients, carers, and medical professionals. Is this the case for you?
12. What are your views on the quality of the health-related content on Wikipedia?

### Data Analysis

A free-text section of the questionnaire was included and analyzed with the interview content. All interviews were transcribed verbatim. The transcripts were analyzed according to grounded theory realist analysis [44,45]. Grounded theory is a well-documented method to explore a concept within a specific context.

Transcripts were systematically coded line-by-line by NF until initial descriptive labels of motivations emerged. These labels were clustered to form a set of concepts that informed the coding paradigm. The new emerging codes were constantly checked retrospectively and prospectively against the higher order categories through constant comparative method [45,46]. NF’s reflections were noted as memos, which also included instances of negative cases [44]. A sample (6/32) of interviews were independently coded by HP and 2 other health psychology researchers and no disagreements were noted.

## Results

### Summary

Almost half of the participants (47%, 15/32) reported currently working in a health-related field, generally as physicians. Of these, 6% (2/32) indicated that their edits were exclusively health-related. The rest of the health-related employment fields covered lung cancer research, health education, health psychology research, regulatory affairs, medical literature, chemistry research, pharmaceutical industry, and health-related advertising. Participants who indicated they were not currently employed in a health-related field (53%, 17/32) did not usually list their professions, but those who were interviewed worked in fields such as engineering, theology, literature, and a couple of participants were students (Table 2).

The reported edits for health-related pages ranged from 1 to more than 50,000. Edit counts do not necessarily reflect the types of edits performed. Of the sample, editorial activities were reported as 16% (5/32) performing primarily major edits, such as adding content and providing quality references, whereas 28% (9/32) reported performing primarily minor edits described as “maintenance issues” that included reverting vandalism, correcting errors, paragraphing, grammar and style, linking articles, checking sources, and simplifying prose. In all, 56% (18/32) reported performing both types of edits. The edit category was self-selected on a demographics questionnaire by respondents and further details provided in the free-comment section.

Country of residence included the United States (n=12), United Kingdom (n=6), Canada (n=4), Australia (n=2), and 1 each in Sweden, The Netherlands, France, Austria, Malaysia, South Africa, and Columbia.

A substantial proportion reported speaking more than 1 language (66%, 21/32), which included French (n=11), Spanish (n=6), German (n=4), Dutch (n=2), Swedish (n=2), and 1 participant each for Chinese, Italian, Afrikaans, Malay, and Bengali. Of the multilingual sample, 25% (8/32) also reported editing Wikipedia pages in these languages.

Approximately 90% (29/32) of Wikipedians were also active contributors in domains other than health, which included both very specific and general descriptors related to topics such as religion, languages, literature, history, sport, politics, architecture, engineering, pop culture, geology and mythology.

### Emergent Categories

People contribute to Wikipedia in different capacities and for various different reasons. The method of realist grounded theory allowed for the emergence of an explanatory theoretical framework. Three interlinked themes that arose from the data were identified and labeled as “help,” “education,” and “responsibility.” Each of these comprised a set of subcategories, shown in Figure 1. In addition, 2 further categories namely “personal fulfillment” and “attitude toward Wikipedia” had a significant motivating quality and were included in the resulting model.

The 3 core motivational systems result in a motivated behavior: contribution to Wikipedia. Contribution, which results in knowledge building or knowledge growth, is also Wikipedia’s core mission and concept as “sum of all knowledge.”

**Table 2:** Participant characteristics (N=32).

Variable		n (%)
**Gender**		
	Male	31 (97)
	Female	1 (3)
**Age (band)**		
	10-20	4 (12)
	21-30	6 (19)
	31-40	11 (34)
	41-50	4 (12)
	51-60	7 (23)
**Highest level of education**		
	Secondary school	3 (9)
	Still at school	3 (9)
	College/university degree (eg, BSc, BA)	8 (25)
	Studying as an undergraduate student	2 (6)
	Master’s university degree (eg, MA, MSc)	6 (19)
	Doctorate/professional degree (eg, PhD, MD)	10 (31)
**Employment**		
	Yes-full time	20 (62)
	Yes-part time	5 (16)
	No	6 (19)
	Retired	1 (3)
**Currently working in a health-related field**		
	No	17 (53)
	Yes	15 (47)
**Wikipedia editing history**		
	<6 months	1 (3)
	<1 year	3 (9)
	1-2 years	5 (16)
	3-5 years	8 (25)
	5-8 years	10 (31)
	≥8 years	5 (16)
**Health-related pages edited**		
	<10	5 (16)
	Approximately 10-20	5 (16)
	Approximately 20-30	1 (3)
	Approximately 30-50	2 (6)
	Approximately 50-100	5 (16)
	>100	14 (44)
**Types of edits**		
	Mainly minor	9 (28)
	Mainly major	5 (16)
	Both	18 (56)

### Motivation Help

All Wikipedians in the study shared a goal of using their skills in order to improve Wikipedia. This was expressed with phrases such as maintaining, ensuring, providing, building, removing, taking care of, adding, clearing, weeding, simplifying, verifying, sharing, expanding, fixing, and helping. These activities can be understood as encompassing both major and minor edits.

There was an almost unanimous answer to the question about what propelled the participants to begin editing Wikipedia, shown in this example:

I found a mistake and I discovered that I could fix it. That has propelled most of my work on the site since then.

It was observed that the motivation help generally stemmed from a sense of importance and care that is also a characteristic of responsibility. In some cases, caring in turn implied being mindful, not just of the self in relation to Wikipedia, but also the relationships others may have with Wikipedia:

I’ve always liked the idea of being able to fix something and have many people be able to benefit from my efforts. I noticed that the...article needed attention, and since I knew about it, I thought I’d tackle the job of fixing it up. No one else seemed like they cared, but the article was read 500-600 times per day, and this bothered me.

I will frequently work on 1 page and make hundreds of edits to it until it is brought up to the professional standard. That is where my prime activity is making major edits to significant disease-related articles. So, this last month I made about 500 edits on the article on...updating it to the most recent literature and I am working in collaboration with a group called translators without borders, to translate these key articles to as many other languages possible.

Not all reported having health-related interests. Their editing behavior was expressed accordingly:

I edit mainly to remove vandalism...therefore, I edit whatever comes up in the queue, which may or may not be health related.

Health-related pages in particular are frequently written from a perspective of a physician rather than a layman. They also require more reliable sources. I try to help solve both of these issues.

Help was a pervasive motivation shown through a number of different editing behaviors. For a very small proportion of Wikipedians, motivation help was not implicit, but was expressed instead as activity reflective of their inherent traits of character:

I am a proofreader by vocation. I cannot leave bad grammar go uncorrected.

### Motivation Education

The motivation education was the most frequently emerging motivation. The initial decision to come to Wikipedia was reported as “intellectual curiosity,” such as a need for information and a need for learning:

I just want to educate myself because I am interested and then share it with people around me.

My edits were sustained by simple interest in topics; I had read a book or article on something and ended up on the Wikipedia page so I’d add a summary or comment about said topic.

In the process of editing a Wikipedia article, the contributors are expected to follow and adhere to Wikipedia’s guidelines, such as verifiability [47], neutral point of view (NPOV) [48], and the guidelines on provision of the evidence-based claims [49]. The process of actively looking for reliable sources has been described as educational:

...very quickly when I began editing Wikipedia I learned that I needed to adjust how I write because there is no argument from authority. It’s all about the citations and sources that you can cite. So the verifiability of the Wikipedia was actually educational for me because I realized that sometimes I said things for which it was hard to find a reference.

The egalitarian nature of Wikipedia, coupled with the guidelines on provision of the evidence-based claims, makes it expected for everyone to support their contributions with a reliable source, which is inherently an educational activity. The process of acquiring new knowledge leads to a better understanding of a specific topic which can be perceived as a form of personal gain (ie, personal fulfillment, which can also be applied across backgrounds):

I usually start by finding the most recent review articles in the medical literature that discuss this topic. Almost invariably, I learn things about conditions that I had previously been unaware of; I can think of a number of examples where my management of patients with a particular condition has been better because I had worked on the relevant Wikipedia article.

As an online knowledge repository, Wikipedia is also a place for groups of people to exchange information. A number of Wikipedians reported that Wikipedia’s international community added to their educational awareness:

Occasionally when I need to look up a rare disease, I’m almost afraid to look at the talk pages because one finds actual patients and their families pleading for help that isn’t there yet...however, it’s important, in fact, essential reading for the researchers working to find treatments for these conditions.

The motivation education was ubiquitous for Wikipedians in the study and encompassed both teaching and learning, either in the process of reading Wikipedia, updating Wikipedia, or through intellectually challenging debates. The motivation education is cyclical and an inevitable state of the editing process on Wikipedia. In sum:

...the short answer to your question [of main motivation] is hedonistic intellectual enjoyment coupled with a sense of responsibility.

### Motivation Responsibility

The motivation responsibility was very closely tied to all other motivations, but the strength of responsibility depended on the attitude toward Wikipedia (ie, beliefs about Wikipedia and beliefs about health care). The goal of many of the contributors in this study was to communicate clearly presented and verifiable information to the world and that applied to the task for both major and minor contributors:

...as a physician, we take the Hippocratic Oath. We try to do the best we can for patients and I consider my patients to be all people globally. And to help all people globally, one way to do that is to provide them access to high quality health care information. So if I can’t see them personally in my emergency department, I know that hundreds of millions of them are looking at Wikipedia to help answer their questions.

I wanted to help the potential future readers who will consult those articles before or instead of a doctor. Their health care decisions may depend on the information they find. I’m no doctor, but at least I can make the articles easier to read.

Several contributors recognized Wikipedia’s scale of influence as the largest repository of online health information, accessible to the whole world, the successful delivery of health care is of vital importance:

Wikipedia is necessarily a distillation of many facts to the things that are most important and valuable. So participating in that is something I felt almost an obligation...I often have felt that everybody in the world should have access to the information and Wikipedia was one place that everyone could access and I couldn’t think of another reference work that would be so useful.

Thus, many Wikipedians reported taking on the responsibility of educating the public. Similarly, they felt responsible to educate their peers, family, friends, students, or colleagues, but were often met with resistance, perhaps something that further strengthened their sense of responsibility:

I want the material to be as accurate as possible so more people will use it. When colleagues denigrate my participation, my response is “If you find something inaccurate on Wikipedia, then you have an obligation to correct it!” I can’t imagine how this could be more important than on a health-related page.

Participants also felt responsible ensuring that the information was reliable and that people adhered to Wikipedia’s editing guidelines. A number of people expressed concern about whether edits maintained an NPOV when concerning controversial topics. Exposed were also instances of when Wikipedia was used as a platform to promote an idea far beyond acceptance in the scientific community:

For vandalism and tendentious editing, my motivation is to maintain the integrity of Wikipedia. Vandalism irks and annoys me, but tendentious editing tends to infuriate me.

...it makes me worried that instead of verifiability and notability being the driving things, it will be other agendas being pushed and that’s disturbing. But I don’t think Wikipedia is going away so I edit it. I continue to edit it because I think it must exist, it is important. And I just wish more people would edit it.

### Attitude Toward Wikipedia

Participant believed in the importance of building health-related content on Wikipedia:

Wikipedia is a beautiful, noble concept.

The interviewed sample attributed strong positive beliefs to Wikipedia, which was identified as a strong motivating factor:

I use it. I support it. It’s a thing worth doing. Wikipedia is a creation of lasting value.

Thus, Wikipedian participants’ beliefs and attitudes about Wikipedia were recognized as influencing their motivations and essential for the process of building Wikipedia. Despite the varying degrees of editorial skills and training of the participants, it became apparent that the interrelated motivations, which can be viewed as value systems, resulted in a motivated behavior model (see Figure 1).

### Personal Fulfillment

Participants derived varying personal benefits in the process of editing health-related pages. Most proclaimed Wikipedia editing as being a hobby; others described it as fun, relaxing, engaging, and rewarding

I find it therapeutic. Yesterday I ended [up] writing an article rather than doing other things which are at the top of my to-do list.

I felt slightly congratulated...so I think such things could be a positive motivator, seeing your work recognized in some form or description.

### Negative Experiences

Dedicated contributors reported engaging in debates or discussions either within their collaborative Wikipedia group (known as Wiki Projects) or over the article’s discussion Talk pages, where both the contributors and readers frequently share experiences.

Their experiences varied but a proportion of the sample expressed being met with hostility and that was particularly relevant to controversial topics:

...it actually happened 1 or 2 times that it was quite aggressive and I lost my motivation for dealing with Wikipedia altogether.

A number of interviewees expressed that they were not welcomed to the Wikipedia community despite their genuine intentions to contribute:

I had a bad experience with Wikipedia...but there is a culture associated with it in that the information has to be added in a certain way. So in good faith I added the content, it wasn’t appropriate and there’s a bit of rudeness on Wikipedia. People were rude to me about what I was trying to do because I did it incompetently. Looking back, I know what I did wrong and I did something inappropriate that was against the community rules but I was discouraged from contributing regularly at that time.

This was also expressed in relation to how Wikipedia accounts are used. There was a divide in opinions about using accounts anonymously. Some emphasized openness because it made it easier for them to trust one another:

When people are hiding behind anonymity, they become a lot less nice. And on Wikipedia we already have a significant issue with civility problems.

However, others expressed that anonymity is necessary to maintain the integrity of Wikipedia:

If I use my authority, then if my edits were wrong they might be accepted because I am in a position of authority, and that would be the opposite of a meritocracy. I think truth is the thing that should trump everything else, which means that authority has no place.

## Discussion

### Value System and Intrinsic Motivation

Results were largely congruent with previous studies on Wikipedia in general, with some notable deviations. As in other studies [20-22,50] the vast majority of our participants were male, in full-time employment, and had obtained a university degree. However, we found a higher mean age and a substantially higher proportion of professionals (PhD, MD) compared to general studies of Wikipedians. We found broadly equal proportions of health specialists and laypeople in our sample.

To explain the underlying motivational drives, we found a process of interlinked value systems, compatible with the results of Kuznetsov [35]. There were 3 overarching primary motivation categories common to all contributors. These were expressed as education, which merged with the responsibility for maintaining accuracy (help), which merged with a sense of obligation (responsibility). These 3 overarching categories of motivations can be understood as self-efficacy as in Social Cognitive Theory [51] and support Kankanhalli and colleagues’ [25] findings in which self-efficacy was found to be the most important predictor of knowledge-sharing behavior in online repositories. Furthermore, the primary overarching motivations were reported as inherent drives of the self, akin to the internal self-concept motivations proposed by Leonard et al [36]. This implies that Wikipedians are motivated to share knowledge because the process resonates with their internal values and beliefs.

It became apparent that motivated behavior arose not only from one’s perception of the self, but also from the underlying beliefs about Wikipedia. Previous studies confirm that the degree of knowledge-sharing behavior significantly relates to individuals’ perceptions of the context in which knowledge is shared [21,37]. Models such as the theory of reasoned action, the theory of planned behavior, or the technology acceptance model that are used to explain individual behavior equally recognize that attitude is crucial in knowledge-sharing intention and behavior [26,38,52].

A recent survey of Wikipedians indicated that 69% contribute to Wikipedia because of the ideology and 60% because they think it is fun [22] supporting the notion that positive outcomes are significant predictors of knowledge-sharing behavior [27] and supporting personal fulfillment as last of the emergent categories.

This study recognized that Wikipedians’ differing editorial roles that can be understood as different levels in terms of “figure/ground” organization [52]. In other words, the ubiquitous motivation help was recognized as the processes of building and maintaining content expressed through various editorial activities of equal importance. This poses a challenge to the current view of Wikipedia in terms of knowledge sharing because not all Wikipedians engaged in knowledge sharing but instead in maintenance activity.

### Implications

The aim of this study was to describe the characteristics of Wikipedians who edited health-related pages on Wikipedia and to gain an understanding of what drives them to contribute to Wikipedia’s health-related pages. This study was the first qualitative exploration of Wikipedia contributors’ motivations, not just in the health context but overall. With the exception of Yang and Lai [21,30], no integrated motivational model has been proposed to explain volunteer contributions in context of Wikipedia. Through the inductive process of grounded theory, no prior theoretical frameworks were “forced” on the data, allowing for the emergence of a realistic depiction of a concept directly from the data. The grounded theory analysis mapped a social process (depicted by Figure 1) of contributing knowledge on Wikipedia driven by individual’s interrelated value systems.

The method of grounded theory also revealed an additional finding, hostility, with a possible connection to Wikipedia accounts’ anonymity (a divide between the editors whose contributions are anonymous and those who, in part or in full, disclose their identity). Although these findings are suggestive rather than definite, they raise a challenge of whether Wikipedia’s philosophy of equality is directly linked to anonymity. Wikipedia is egalitarian, a place where everybody are peers and a place where everyone has an equal right to edit contents. According to our results, a portion of Wikipedians believe that nonanonymous accounts would aid in civility, but also that nonanonymous accounts may create a hierarchy, a structure contradictory to Wikipedia’s egalitarian philosophy.

This study also provided new evidence regarding the contributory behavior of Wikipedians: participants engage in contribution by utilizing their skill and not necessarily through knowledge sharing. Recently, a term was coined which describes Wikipedia contributors as “knowledge philanthropists” [53]. Although this term applied to a proportion of participants in this study, it is not applicable to all, particularly those who do not contribute to but instead “maintain” Wikipedia’s content. Our broader view serves to recognize that everyone can contribute to Wikipedia without necessarily requiring expert knowledge.

### Limitations

The sample of 44 articles used in the study may not be a representative sample of all health-related articles available on Wikipedia. The articles were randomly sampled from a total of approximately 3000 keywords complied from 3 medical databases and Portal Medicine’s Featured Articles. An alternative approach would be to manually compile a list from Wikipedia’s Category:Health, but the list would still not include all biomedical and drug-related articles.

Sampling bias may also apply to the recruitment of contributors. Selecting the most recent 5 contributors posed issues because some users appeared in the most recent 5 in more than 1 sampled article. In these instances, the researcher skipped accounts already contacted and contacted the next account down the list. This suggests that the editorial population of health content on Wikipedia is small. Another approach would be to select contributors according to the number of edits performed, although this may prove difficult because the numbers of edits are not necessarily indicative of editor’s activity or the type of editorial involvement.

The response rate for the questionnaire was relatively low, for which the reasons may have been the mode and duration of the advertisement of the study. Only 32 participants completed the survey and 17 were interviewed. This is only a sample and does not represent all Wikipedians active on health-related articles. (We note the list of participants in WikiProject Medicine is much larger with 424 members as of August 2014 [54].) We suspect that this is a reflection of recruiting people via their Wikipedia user pages, which means participants had to be active on Wikipedia during the limited study period to see the recruitment message. It is fair to assume that the identified motivations might be sufficiently pervasive to be represented in a small sample of Wikipedians; however, varying levels of editorial skill and knowledge are not likely to be sampled deeply enough to be representative. The sample were recruited in a specific time frame and results may not be applicable over time. There are currently still challenges with increasing participation in contributing to Wikipedia health-related content. Some initiatives are already in place, such as the Translation Task Force and Wiki Project Med Foundation, a Wikipedia education program designed to educate medical students about the process and value of contribution to Wikipedia health pages, as well as also collaborating with a number of organizations including the Cochrane Collaboration, Cancer Research UK, and the National Institute of Health [55].

The success will largely depend on user’s satisfaction and recognition of the potential benefit that can be gained from such editorial activities. By understanding Wikipedians’ motivations for editing health-related content, we can better recruit more people to the task. Equally important is recognizing the factors that may discourage people and more specifically professionals, from contributing to Wikipedia. Characterizing editing behavior and editors also allows us to understand the processes underlying Wikipedia’s health-related content.

## Abbreviations

IP: Internet Protocol
NHS: National Health Service
NORD: National Organization of Rare Disorders
NPOV: neutral point of view
